# The burden of atopic dermatitis in Portuguese patients: an observational study

**DOI:** 10.1038/s41598-024-55965-y

**Published:** 2024-03-02

**Authors:** Pedro S. Coelho, Miguel Apalhão, Guilherme Victorino, Cristina Cardoso, Joana Camilo, João Maia Silva

**Affiliations:** 1 NOVA Information Management School (NOVA IMS), Universidade NOVA de Lisboa, Lisbon, Portugal; 2 Dermatology Department, Hospital de Santa Maria, Centro Hospitalar Universitário de Lisboa Norte, Lisbon, Portugal; 3 Dermatology Research Unit, Instituto de Medicina Molecular, University of Lisbon, Lisbon, Portugal; 4 SANOFI PORTUGAL, Lisbon, Portugal; 5 ADERMAP-Atopic Dermatitis Association, Lisbon, Portugal; 6 Dermatology Center, Hospital CUF Descobertas, Lisbon, Portugal

**Keywords:** Diseases, Skin diseases, Pruritus

## Abstract

Atopic dermatitis (AD) is a common inflammatory skin condition that significantly affects patients' lives and imposes both economic and non-economic burdens. The precise societal and individual consequences of AD remain incompletely understood. This study aimed to characterize AD in Portuguese patients and assess its personal, familial, and societal implications, including health status and quality of life. The research, conducted from June 2019 to January 2020, involved 204 confirmed AD patients in Portugal, who completed a 70-question questionnaire. Results show that, on average, patients experienced a two-year delay in diagnosis, with two-thirds having allergic comorbidities. Late-onset AD (after age 20) was found to be correlated with worsening symptoms post-diagnosis. Globally, patients reported substantial effects on health, quality of life, and mental well-being. Effects include significant levels of anxiety, frustration and sleep disorders. Severe AD correlated with more suffering and reduced perceived health, indicating a link between disease severity and quality of life. Remarkably, despite questionable effectiveness, 92% of severe AD patients were prescribed antihistamines, while only 19% received biological treatments. In Portugal, delayed AD diagnosis hinders timely treatment, and despite its profound impact and high comorbidity rates, AD patients tend to remain undertreated. Recognizing the personal and societal repercussions is crucial for enhancing care, contributing to improving QoL, social functioning and global well-being.

## Introduction

Atopic dermatitis (AD) is a Th-2 mediated inflammatory disease defined by chronic and relapsing skin inflammation, a significant imbalance of skin barrier function, and intense pruritus^1,2^. AD first manifests during early childhood in most cases, but up to 25% of adult patients declare the onset of their dermatosis in adulthood^3^. Persistence of childhood-onset AD into adulthood is common, occurring in about half the cases.

The incidence and prevalence of AD have been a matter of discussion and seem to vary according to geographic location and time period^4^. The estimated worldwide prevalence of AD ranges from 1 to 3% in adults to 20–25% in children^5^. However, it may afflict up to 7.2% of adults in Western countries^6^.

The impacts and burden of AD on patients far exceed the appearance of skin lesions. Indeed, patients with atopic dermatitis are at increased risk of infections and other allergic comorbidities (such as asthma, rhinitis, and eosinophilic esophagitis). They also show severe sleep disturbances, increased rates of anxiety, depression, and suicidality. Recent studies have shown an association between AD and auto-immune diseases and cardiovascular diseases^7,8^.

It comes as no surprise that AD carries significant curtailments in quality of life^9^, which may translate into significant daily life disruption for patients and families alike^10–12^. The impact of AD on sleep quality, the disruptive consequences of chronic intense pruritus, the psychological consequences secondary to self-image compromise and limitations of daily activities to avoid aggravating triggers have been shown to lead to labour and educational absenteeism. These impacts also provoke presenteeism (defined as the loss of productivity even when the patient is present at work/school), in addition to the direct and indirect costs related to healthcare resources consumption^13–16^. Data on these aspects are sparse in Europe, thus limiting public health and regulatory decisions on the cost-effectiveness analysis of innovative therapeutic drugs.

Herein, we present the results of a survey aiming to characterize the disease, quantify social, family and individual burden of AD and treatment strategies in the Portuguese reality. This paper will focus on the self-reported impacts of AD in affected patients and the characterization of their disease history.

## Methods

This observational study was conducted on patients who reside in Portugal with a confirmed AD diagnosis. Only patients with a previous diagnosis from a specialist (physician) were included in the study. Diagnostic by physicians includes criteria as pruritus, morphology and distribution, personal and family history, chronology of symptoms, among others. Population included both adults and non-adults. Two hundred and four patients filled in a specifically designed questionnaire with 70 questions between June 2019 and January 2020 for the study. The design of rating instruments was carried out in close collaboration with specialists in AD and based on the integration of metrics validated in previous studies (Dermatology Life Quality Index—DLQI^32^, EQ-5D^33^ scales, including Visual Analog Scale-VAS). Both scales have been previously validated into Portuguese^34,35^. The sample was obtained by addressing the whole database of patients enrolled in the national association of atopic dermatitis (ADERMAP). These patients were also encouraged to resent the questionnaire to other patients not enrolled in the association. Due to this fact, the sample includes non-probabilistic components.

### Data collection

Data on sociodemographic characteristics, disease characterisation, emotional and social impact and treatment strategy, including quality of life (DLQI and EQ-5D scales), were collected. Patients answered the questionnaire considering the previous 12 months.

Data were collected through online surveys, with the collaboration of the patient association ADERMAP and a sample of dermatologists to disseminate the questionnaire to patients. All participants provided informed consent. In order to increase response rate and mitigate possible non-response bias the data collection used up to 3 callbacks to non-responds.

### Statistical analysis

All continuous variables are expressed using means, and categorical variables are expressed using proportions. Between-group analysis was performed using t-tests for differences. The χ^2^ test was used to test the association between categorical variables.

Multiple group comparisons were performed using ANOVA models.

In order to control and mitigate possible sampling and non-response biases the sample was post-stratified by disease severity (3 levels- mild, moderate and severe) using known distribution at population level.

## Results

### Demographic characterisation

A total of 204 individuals responded to the questionnaire. Most respondents (64%) were female, and the average age was 31.1 years old. Female weight is compatible with previous published results regarding different geographies^31^. The sociodemographic characteristics of this population are explored in Supplementary Table 1.

### Disease characterisation

#### Duration of disease and time of diagnosis

**Figure 1:**
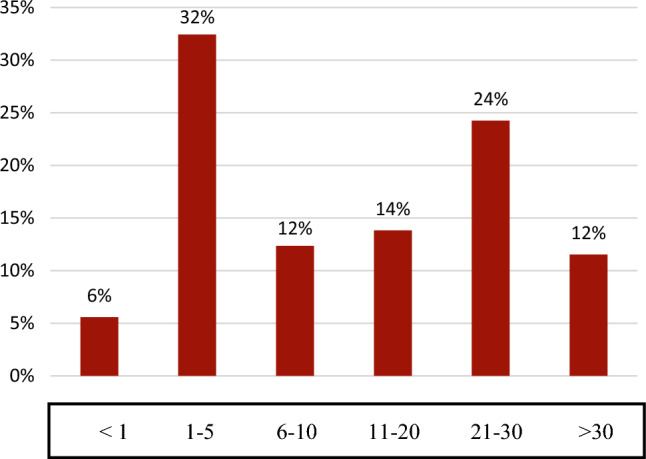
For how long have you had the diagnosis of atopic dermatitis? (In years).

The average duration of the disease was 15 years. Half the respondents have suffered from AD for more than ten years, while 36% had complaints for more than 20 years. (Fig. 1).

Only 22% of the individuals reported the first symptoms of AD after 20, and 57% of the participants experienced the first debilities before the age of 10.

The average time to diagnosis was 2 years, with one-third of the patients receiving their AD diagnosis within the first six months after the onset of symptoms, while 29% of the patients report a delay of 1 year or longer until the diagnosis of AD was established. In 10% of the cases, the diagnosis was only established more than 6 years after the first symptoms of the disease. In 19% of the cases, the participants could not tell the precise chronology of their diagnosis but reported it had been known since childhood.

A Dermatologist made the AD diagnosis in 59% of the cases, followed by a Pediatrician, Family Doctor, or Allergologist in 17%, 14% and 10% of the participants, respectively.

#### Affected body areas

The upper limbs were affected in more than half the respondents. Head and face were affected in 39% of the cases. Lower limbs were also reported as being affected in 38% of the participants. Genital lesions were reported in 17% of the cases, and 6% of the participants declared a generalised distribution of their dermatosis.

Patients were classified into 3 severity levels based on self-report. Detailed explanations produced by dermatologists about what characterizes a mild, moderate and severe form of atopic dermatitis were offered to the patients, who were asked to select the category that better fit their condition. Cross-checks were also performed by asking in a later stage of the questionnaire about the prevalence of specific symptoms. The distribution of dermatosis seems to vary depending on its severity. While no participants with mild AD mentioned foot involvement, in severe cases, this area was reported to be afflicted in 17% of the patients (p < 0.001). This difference was also noted in head and face lesions (17% in mild cases vs 52% in severe cases, p = 0.003), lower limbs (25% vs 61%, p = 0.007), abdomen (8% vs 38%, p = 0.001), and generalised distribution (8% vs 25%, p = 0.056). Less significant and statistically non-significant differences were reported in arms and forearms (50% vs. 64%, p = 0.356), hands (42% vs 36%, p = 0.689) and genitals (17% vs. 19%, p = 0.862) (Table 1).

**Table 1:** Prevalence of lesions per anatomic region, according to self-reported severity of the disease.

Anatomic region	SEVERITY			Overall (%)	p-value (severe-mild)
	Mild (%)	Moderate (%)	Severe (%)		
Head and neck	17	66	52	39	0.003*
Hands	42	59	36	49	0.689
Feet	0	7	17	3	0.000*
Arms	50	63	64	56	0.356
Legs	25	53	61	38	0.007*
Thorax and abdomen	8	28	38	18	0.001*
Back	8	24	40	16	0.000*
Genitals	17	17	19	17	0.862
Generalised	8	2	25	6	0.056
Other	33	2	4	19	0.035*

Statistically significant differences in prevalence between mild and severe per location are signalled with * for the reader’s convenience.

#### Severity of disease

Most patients (54%) reported mild disease, while 43% considered their disease moderate in severity, and only 3% complained of severe AD. About 37% of patients consider their disease to have improved since diagnosis, 35% report progressive worsening, and 18% think their disease to have remained stable in severity since the diagnosis.

Association between the progression of AD and age of onset of first symptoms was found significant (p < 0.0001). In patients who presented the first symptoms before the age of 10, 50% reported stable disease, and 37% reported progressive improvement of the complaints over time. Of the individuals for whom AD first presented between the ages of 11 and 20, 76% reported stable disease. Only 6% and 17% of patients considered their disease to have improved or worsened, respectively, over time. The older onset of disease (older than 20) seems to increase the propensity to progressively worsening AD severity over time (70% of the cases) (Table 2).

**Table 2:** How has your atopic dermatitis progressed over time since diagnosis?

Onset of symptoms	Progression of AD		
	It has improved over time (%)	It has been stable over time (%)	It has worsened over time (%)
< 10 yo	37	50	13
11–20 yo	6	76	17
> 20 yo	15	15	70

#### Comorbidity conditions

Two-thirds of the participants reported clinical symptoms of allergic diseases. Among these patients, rhinitis was found in 72% of the cases, followed by asthma (37%), food allergy (31%) and a previous anaphylactic episode (2%).

Cardiovascular disease was reported in 2% of the cases, and 29% denied any known medical condition other than AD.

### Social impact of AD

#### Impact on Quality of Life (QoL)

Half the patients scored 5 or above on an analogical scale for suffering (1–10), and 20% scored 7 or higher. The mean score was 4.4. The subjective suffering seems to correlate with AD severity, as patients with mild disease scored on average 3.0, while those with moderate AD scored 5.8 and those with severe AD scored an average of 7.9. The effect of AD severity was significant (p < 0.0001).

On the Dermatological Life Quality Index (DLQI), 73% of participants scored 6 or higher, while 36% scored above 11. The average score was 9.4 (Table 3), with significant differences across disease severities: patients suffering from mild disease scored an average of 6.6. In contrast, moderate cases scored 12.3 on average, and patients suffering from severe AD had a mean score of 18.7. As expected, differences between groups are found to be statistically significant(p < 0.0001).

**Table 3:** Means and standard-deviations for DLQI across severity levels.

Severity	Mean	Standard deviation
Mild	6.6	3.2
Moderate	12.3	5.6
Severe	18.7	5.7
Global	9.4	5.5

The impacts on QoL are associated with factors (Table 4), but three factors seem to be particularly relevant: the frequent feeling of pain, itching or stinging, found in 61% of the participants; the frequent feelings of shame due to skin lesions, reported by 41% of the patients; and the high impact the skin lesions had in clothing choices, reported by 40% of the respondents.

**Table 4:** Impact of atopic dermatitis on patients’ lives.

Over the last 12 months…	A lot (%)	Significantly (%)	A little (%)	Not at all (%)	Not applicable (%)
Have you felt pain, itching, or stinging on your skin?	17	44	29	5	6
Did you feel embarrassed or ashamed due to your skin condition?	14	27	39	17	2
To what extent did your skin condition impact your daily activities (shopping, house chores, etc.)?	5	22	35	22	16
To what extent did your skin condition influence the clothes you chose to wear?	18	22	25	24	11
To what extent did your skin condition affect your social and leisure activities?	5	21	28	39	6
To what extent did your skin condition affect your physical and sports activities?	6	16	34	26	18
Did your skin condition cause relationship issues with your partner, family or friends?	1	6	17	69	7
To what extent did your skin condition affect your sexual life?	2	4	19	58	18
To what extent has the treatment for your skin condition been an issue in your life, e.g. Staining your clothes, taking too much time, etc.?	6	13	51	28	3

Additionally, we tested the possible existence of a relationship between the DLQI score and sociodemographic characteristics of patients, including gender, age class, education, and income. We used an n-way ANOVA controlled by disease severity (mild, moderate, severe). Assumption of residual normality was confirmed using Kolmogorov–Smirnov test (p > 0.08). We did not find statistically significant effects from age, income, or education. Nevertheless, the age metric proved to be significant (p < 0.001) with a pattern of DLQI increase with age, with the 65 years or older patients having a DLQI 6 points higher than patients of the same severity class with less than 25 years old.

Overall, AD is a relevant factor in sleeping problems (in 34% of the participants), anxiety (33%) and feelings of frustration (45%). Indeed, sleep disturbance seems to be marked, as 36% of patients report three or more nights a month with poor sleep quality/quantity, and 8% report more than 14 such nights each month.

On an analogical scale for Overall Health status (0—Worst Overall Health Possible; 100—Best Overall Health Possible), these patients scored an average of 75, with 39% of patients scoring 75 or less. Patients with severe AD had significantly lower average scores (56) than those with moderate (66) or mild (83) disease (p < 0.0001).

Patients were asked to provide a score on an analogical scale of 0–100 to quantify the impact of AD on their QoL (0—no impact; 100—very high impact). An average score of 51 was reported, with 52% of patients reporting a score higher than 50 and 21% reporting a score above 75.

### Treatment

About 90% of individuals reported consistent use of emollients and hypoallergenic products for their hygiene. Topical corticosteroids were used over the last 12 months in 66–94% of the respondents, with self-reported mild and severe disease, respectively, and other topical immunosuppressants (e.g. Calcineurin inhibitors) had been applied, respectively, in 11% and 62% of these patients.

Systemic corticosteroids were not taken by patients reporting mild disease but were administered in 29% of the moderate and 66% of severe cases. Cyclosporine was prescribed in the preceding year to 3% of moderate and 34% severe cases, while other systemic immunosuppressants were taken by 6% and 22%, respectively.

While oral antihistamines were taken by 33% to 92% of patients, only 19% of patients with severe disease were prescribed biological drugs for their AD.

Treatment data are summarised in Table 5.

**Table 5:** Treatment regimens undertaken by the patients currently and anytime over the last 12 months, according to self-reported AD severity.

Treatment	Mild (%)	Moderate (%)	Severe (%)	Overall (%)
Which of the following treatments have you been on, anytime over the last 12 months?				
Hypoallergenic creams and/or ointments (emollients, hydrating creams, etc.)	78	93	99	85
Topical corticosteroids	67	87	94	76
Cyclosporin A	0	1	34	2
Other systemic immunosuppressants: such as Azathioprine, Mycophenolate Mofetil, Methotrexate	0	6	22	3
Systemic corticosteroids	0	28	66	14
Topical calcineurin inhibitors	11	59	62	33
Biological drug treatments (Omalizumab, Dupilumab)	0	0	19	1
Oral antihistamines	33	78	92	54
None of the above	11	1	0	7
Other	22	9	12	16
Which of the following treatments are you currently on?				
Hypoallergenic creams and/or ointments (emollients, hydrating creams, etc.)	89	93	88	90
Topical corticosteroids	56	65	75	60
Cyclosporin A	0	1	12	1
Other systemic immunosuppressants: such as Azathioprine, Mycophenolate Mofetil, Methotrexate	0	4	7	2
Systemic corticosteroids	0	10	19	5
Topical calcineurin inhibitors	11	38	38	24
Biological drug treatments (Omalizumab, Dupilumab)	0	0	18	1
Oral antihistamines	33	56	67	44
None of the above	0	1	1	1
Other	0	7	16	4

## Discussion

The present study is the first to extensively explore the self-reported impact of atopic dermatitis across different aspects of patients' lives in Portugal.

These results show that despite being the most common inflammatory skin condition worldwide, atopic dermatitis may not be easy to diagnose. Indeed, the significant delay between symptom onset and diagnosis may lead to significant morbidity and impact quality of life. This delay may be because AD remains a clinical diagnosis. Thus, in a condition with diverse clinical manifestations, expertise is required to assess the patient as a whole and establish the appropriate diagnosis. Data on the average time to diagnosis is scarce in the available literature, but further investigation is paramount to identify the best strategies to overcome these limitations. Our data shows that Dermatologists may be the physicians most acquainted with AD, as these professionals diagnosed almost two-thirds of the patients, despite the accessibility issues that hinder immediate access to these professionals in Portugal in most instances.

Adult-onset AD seems to associate with increasing severity over time. It has been established that risk factors and clinical presentations of adult-onset AD differ from those of childhood AD^17–19^. The classical description of AD as a pediatric condition may delay the correct diagnosis of adult-onset AD, which might be under-recognised and under-reported^20^. Additionally, results support a degradation of quality-of-life (DLQI) with age after controlling for the disease severity.

We also note the high prevalence of other allergic comorbidities self-reported by AD patients in Portugal. These data are concordant with other published studies on the prevalence of rhinitis, asthma and food allergies^21–25^. However, it should be noted that these data are based on self-reported information, and food allergy might be over diagnosed by both patients and physicians alike, as even when prick tests are positive, challenge tests may frequently be negative^26–28^.

Anxiety, frustration and sleep disorders seem to be frequent in the Portuguese AD population, in accordance with the published literature in other geographical areas^9,14^. These perturbations might derive from the significant impact AD represents on everyday life choices, from clothing options to professional decisions. Likewise, it is essential to highlight that AD seems to significantly affect the self-perceived level of healthiness on an analogical scale, with patients suffering from severe AD reporting an average 56/100 overall health, a value translating as poor health when considering a predominantly cutaneous condition.

Survey results tend to support the idea that treatment of AD remains sub-optimal in Portugal. This conclusion results from the observation that AD patients tend to remain undertreated. In fact, systemic steroids are not recommended for the maintenance treatment of AD. It is noted that cyclosporine A is seldom an option for long-time treatment of chronic conditions due to toxicity. Other conventional systemic treatments are used off-label in AD, with significant potential for adverse effects. Biological drugs approved for this indication, along with other atopic comorbidities, are prescribed for a small minority of severe AD cases. On the other hand, oral antihistamines, which have been noted to be of small benefit in AD^29,30^, are prescribed for a significant majority of patients. This prescription profile may be the direct translation of health policies that restrict access to novel therapies that come at a higher cost but may be beneficial from an overall financial point of view if the indirect costs of the disease are tackled by effective treatment.

### Limitations

One major limitation of this study is related to sample size. Although the sample supports the estimation of population characteristics with a good level of accuracy (accuracy for estimating average DLQI is 1.1 points with 95% confidence level), it does not support adequate accuracy for separate estimation by characteristics with more than 3 categories (for ex. some socio-demographic classes with more than 3 categories). Another limitation to stress is the fact that we have used a cross-sectional study, where a longitudinal one would be more adequate to measure evolutions, as for ex. changes in treatment strategy in the course of 12 months. Finally, it is to stress that the measurement was based on self-report. Although this constitutes a limitation, it was mitigated by offering detailed definitions and descriptions to patients, as well as a support line to respondents. Additionally, it is not to be excluded that the online data collection method may contribute to a reduced (or more difficult) response for older and less literate segments of the population.

## Conclusions

AD is a prevalent condition which carries significant impacts on QoL. Correct and timely diagnosis is paramount to improving outcomes, but many patients still experience significant delays in diagnosis. Recognising and addressing comorbidities in AD and its toll on mental and social health is a crucial determinant of improving QoL, social functioning, global well-being, and optimising AD outcomes. Specialists are central to the diagnosis, management, and follow-up of patients with AD and should refer patients to other healthcare providers when necessary to address non-cutaneous comorbidities. The late diagnosis shown by the significant proportion of these patients tends to show that the disease is still of difficult diagnosis by clinicians with long terms impacts over patients QoL. Strategies to treat the disease should not ignore that results support a tendency for undertreatment, particularly for more severe cases. Novel effective therapies should not be relegated to last-line treatment options, and pharmacoeconomic studies are needed to instigate stakeholders to change drug access policies.

### Supplementary Information

Supplementary Table 1.

## Data Availability

The data used in this study are available upon request. Researchers interested in accessing the data can contact the corresponding author (G.V.) for further information (gmvictorino@novaims.unl.pt).
