# Supplementary material for: The burden of atopic dermatitis in Portuguese patients: an observational study
Source: Sci Rep. 2024 Mar 2;14:5181. doi: 10.1038/s41598-024-55965-y (PMC10908846; doi:10.1038/s41598-024-55965-y)
Supplement: Supplementary file 1 — Supplementary Table 1. [file 41598_2024_55965_MOESM1_ESM.pdf]

The Burden of Atopic Dermatitis in Portuguese patients: an observational study

Supplementary Table 1
Sociodemographic characteristics of the respondents, according to self-reported severity of disease

	Mild	Moderate	Severe	Overall
**Gender**				
Female	75%	52%	50%	**64%**
Male	0%	26%	32%	**12%**
Not answered	25%	22%	18%	**23%**
**Age**				
< 25 years old	33%	27%	44%	**31%**
25 - 34 years old	22%	41%	20%	**30%**
35 - 44 years old	33%	21%	29%	**28%**
45 - 54 years old	0%	6%	5%	**3%**
55 - 64 years old	11%	3%	2%	**7%**
≥65 years old	0%	1%	0%	**1%**
Average (years)	32.4	29.7	27.6	**31.1**
**Education**				
No studies/Illiterate	0%	1%	1%	**1%**
Elementary School	11%	9%	7%	**10%**
High School	11%	21%	30%	**16%**
Higher Education	78%	67%	56%	**73%**
Other	0%	1%	5%	**1%**
**Civil Status**				
Single	33%	61%	69%	**46%**
Married	67%	22%	15%	**46%**
Civil Union	0%	14%	15%	**7%**
Divorced	0%	3%	1%	**1%**
Widow(er)	0%	0%	0%	**0%**
**Number of individuals living in the patient's household**				
1	0%	16%	12%	**7%**
2	22%	25%	21%	**23%**
3	44%	38%	23%	**41%**
4	11%	16%	33%	**14%**
5	22%	6%	10%	**15%**
Average	3.3	2.7	3.1	**3.1**
**Number of first-line relatives (parents; grandparents; siblings) with atopic dermatitis**				
None	13%	52%	47%	**30%**
1	25%	35%	30%	**29%**
2	38%	12%	14%	**26%**
3	13%	2%	5%	**8%**
≥4	13%	0%	3%	**7%**
Average	2.0	0.6	1.0	**1.4**
